# Three new macropterous species, and additional records, of *Oedichirus* Erichson, 1839 from China (Coleoptera, Staphylinidae, Paederinae)

**DOI:** 10.3897/zookeys.1280.192656

**Published:** 2026-05-22

**Authors:** Shi-Chao Tang, Wang Xu, Zhong Peng

**Affiliations:** 1 Department of Biology, Shanghai Normal University, Shanghai, 200234, China Department of Biology, Shanghai Normal University Shanghai China; 2 Shenzhen Ecological and Environmental Monitoring Center of Guangdong Province, Shenzhen, 518049, Guangdong, China Guangdong Greater Bay Area, Change and Comprehensive Treatment of Regional Ecology and Environment, National Observation and Research Station Shenzhen China; 3 Guangdong Greater Bay Area, Change and Comprehensive Treatment of Regional Ecology and Environment, National Observation and Research Station, Shenzhen, 518049, Guangdong, China Shenzhen Ecological and Environmental Monitoring Center of Guangdong Province Shenzhen China

**Keywords:** China, key, morphology, new records, new species, rove beetles, taxonomy

## Abstract

Three new macropterous species of the genus *Oedichirus* Erichson, 1839 from China, are described and illustrated: *O. acutus* Tang & Peng, **sp. nov**. (Libo, Guizhou), *O. biwenxuani* Tang & Peng, **sp. nov**. (Yingjiang, Yunnan) and *O. excisus* Tang & Peng, **sp. nov**. (Qujing, Yunnan). *Oedichirus laoticus* Rougemont, 2018 is reported from China for the first time. Additional provincial records are provided: *O. chapmani* Cameron, 1940 from Hubei, Guangdong and Yunnan; *O. longipennis* Kraatz, 1859 from Zhejiang, Guangdong and Hainan; and *O. ovalisis* Duan, Yue & Li, 2024 from Guangxi. A key to the macropterous species from China is provided.

## Introduction

The Asian fauna of *Oedichirus* Erichson previously included 75 described species, exhibiting a high degree of endemism, with 33 macropterous and 42 micropterous species (Rougemont 2018a, 2018b; Assing 2019; Duan et al. 2024). In contrast, the Chinese fauna has received less research attention compared to the Ethiopian fauna (Duan et al. 2024). Following necessary taxonomic revisions—for instance, records of *O. lewisius* Sharp, 1874 from Shanghai and Guangxi have been reassigned to *O. longipennis* Kraatz, 1859 based on Rougemont (2018b)—a total of 16 species were previously confirmed in China, comprising eight macropterous and eight micropterous species, thus reflecting a provisional equilibrium in wing-morph composition (Assing 2014; Li et al. 2015; Rougemont 2018a, 2018b; Xu et al. 2020; Duan et al. 2024).

This paper provides taxonomic and faunistic data for nine macropterous species of *Oedichirus* from China. These include the description of three new species, the first record of *O. laoticus* Rougemont, 2018 in China, and new provincial records for *O. chapmani* Cameron, 1940, *O. longipennis* Kraatz, 1859, and *O. ovalisis* Duan, Yue & Li, 2024. A key to the macropterous species of China is also presented. To date, the total number of *Oedichirus* species known from China has increased to twenty.

## Material and methods

The genitalia and other dissected parts were mounted on plastic slides and attached to the same pin as the respective specimens. Photographs were taken with a Canon EOS 7D camera with a Canon MP-E 65 mm macro lens or with a Canon G9 camera mounted on an Olympus CX 31 microscope.

Abbreviations: Body length (**BL**) from the anterior margin of the head to the abdominal apex; forebody length (**FL**) from the anterior margin of the head to the posterior margin of the elytra; head length (**HL**) from the anterior margin of the frons to the posterior margin of the head; head width (**HW**): maximum width of head; length of antenna (**AnL**); pronotum length (**PL**) from anterior margin of pronotum to its posterior margin; pronotum width (**PW**): maximum width of pronotum; elytral length (**EL**) at the suture from the apex of the scutellum to the posterior margin of the elytra (at the sutural angles); elytra width (**EW**): maximum width of elytra; length of aedeagus (**AL**) from the apex to the base of the aedeagal capsule.

All material treated in this paper is deposited in the Insect Collection of Shanghai Normal University, Shanghai, China (**SNUC**). The type labels are cited in the original spelling.

## Results

### Oedichirus acutus

Taxon classificationAnimaliaColeopteraStaphylinidae

Tang & Peng sp. nov.

E793D8F9-6ECA-51F7-9978-7391419901EB

https://zoobank.org/30FFF2CD-B698-4140-93E0-ADBDC8B7A9B6

Figs 1A, 2

#### Type material.

***Holotype***. China – **Guizhou** • ♂; glued on a card with two labels as follows: “China: Guizhou Prov., Libo Co., Maolan N. R., Bizuo, 25°17'51"N, 106°04'28"E, 679 m, 01.V.2017, Jiang, Hu, Liu & Zhang leg.” “HOLOTYPE: *Oedichirus acutus* Tang & Peng, 2026, new species.” [red handwritten label]; (SNUC). ***Paratypes***. China – **Guizhou** • 1 ♀; glued on a card with two labels as follows: “China: Guizhou Prov., Libo Co., Maolan N. R., Bizuo, 25°17'16"N, 106°04'18"E, 850 m, 26.IV.2017, Jiang, Hu, Liu & Zhang leg.” “PARATYPE: *Oedichirus acutus* Tang & Peng, 2026, new species.” [yellow handwritten label]; (SNUC); China – **Guizhou** • 1 ♂; glued on a card with two labels as follows: “China: Guizhou Prov., Libo Co., Maolan N. R., Dongtang, 780 m, 21.VII.2015, Li-Zhen Li leg.” “PARATYPE: *Oedichirus acutus* Tang & Peng, 2026, new species.” [yellow handwritten label]; (SNUC).

#### Description.

Measurements (in mm) and ratios: BL: 8.44–9.00; FL: 3.78–4.00; HL: 0.78–1.00; HW: 1.12–1.13; AnL: 2.68–2.83; PL: 1.15–1.26; PW: 1.00–1.11; EL: 1.41–1.49; EW: 1.49–1.60; AL: 1.29; HW/HL: 1.11–1.43; HW/PW: 1.00–1.11; HL/PL: 0.68–0.87; PW/PL: 0.82–0.94; EL/PL: 1.12–1.26.

**Figure 1.**
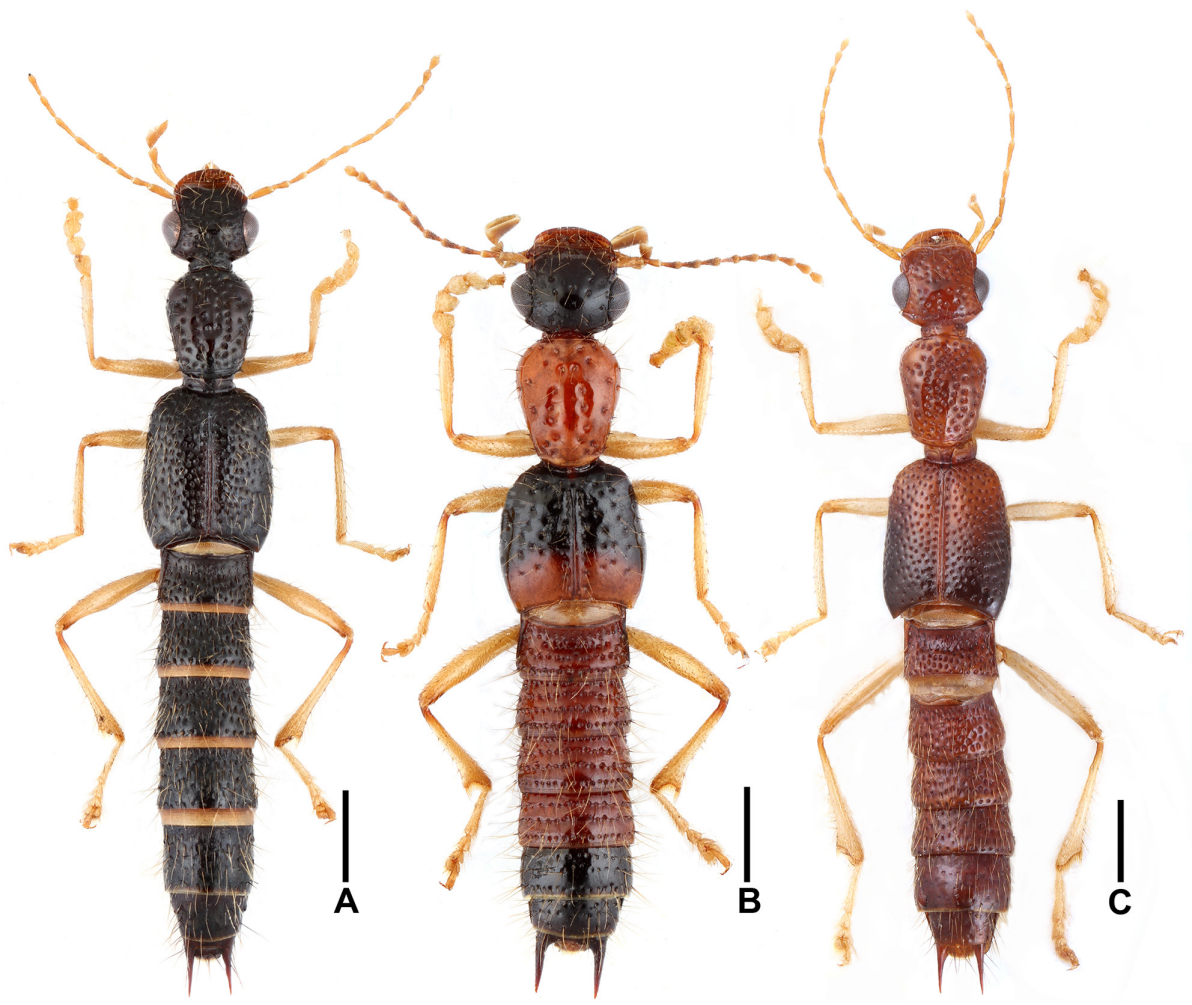
Habitus (**A**–**C**). **A**. *Oedichirus acutus*; **B**. *Oedichirus biwenxuani*; **C**. *Oedichirus excisus*. Scale bars: 1.0 mm.

***Habitus*** as in Fig. 1A. Coloration: body black; antennae and legs yellowish brown.

***Head*** weakly transverse, broadest across eyes. Lateral contours behind eyes converged sharply with basal angles distinct. Dorsal surface with coarse, moderately dense punctures. ***Eyes*** strongly convex, 2.47–2.59 times as long as distance from posterior margin of eye to posterior constriction.

***Pronotum*** approximately as broad as head, widest anteriorly and gently tapering posteriorly. Median dorsal portion slightly convex, surface with irregular coarse punctures, distinctly larger than those on head.

***Elytra*** oblong, surface with dense and coarse punctures, distinctly denser and finer than those on head and pronotum. Humeral angles and wings well developed.

***Abdomen*** with dense punctures, nearly arranged in rows; punctures on segments III–VI weakly coarser and denser than on segments VII–VIII. Interstices with fine and shallow microsculpture composed of transverse striae; posterior margin of tergite VII with palisade fringe.

**Figure 2.**
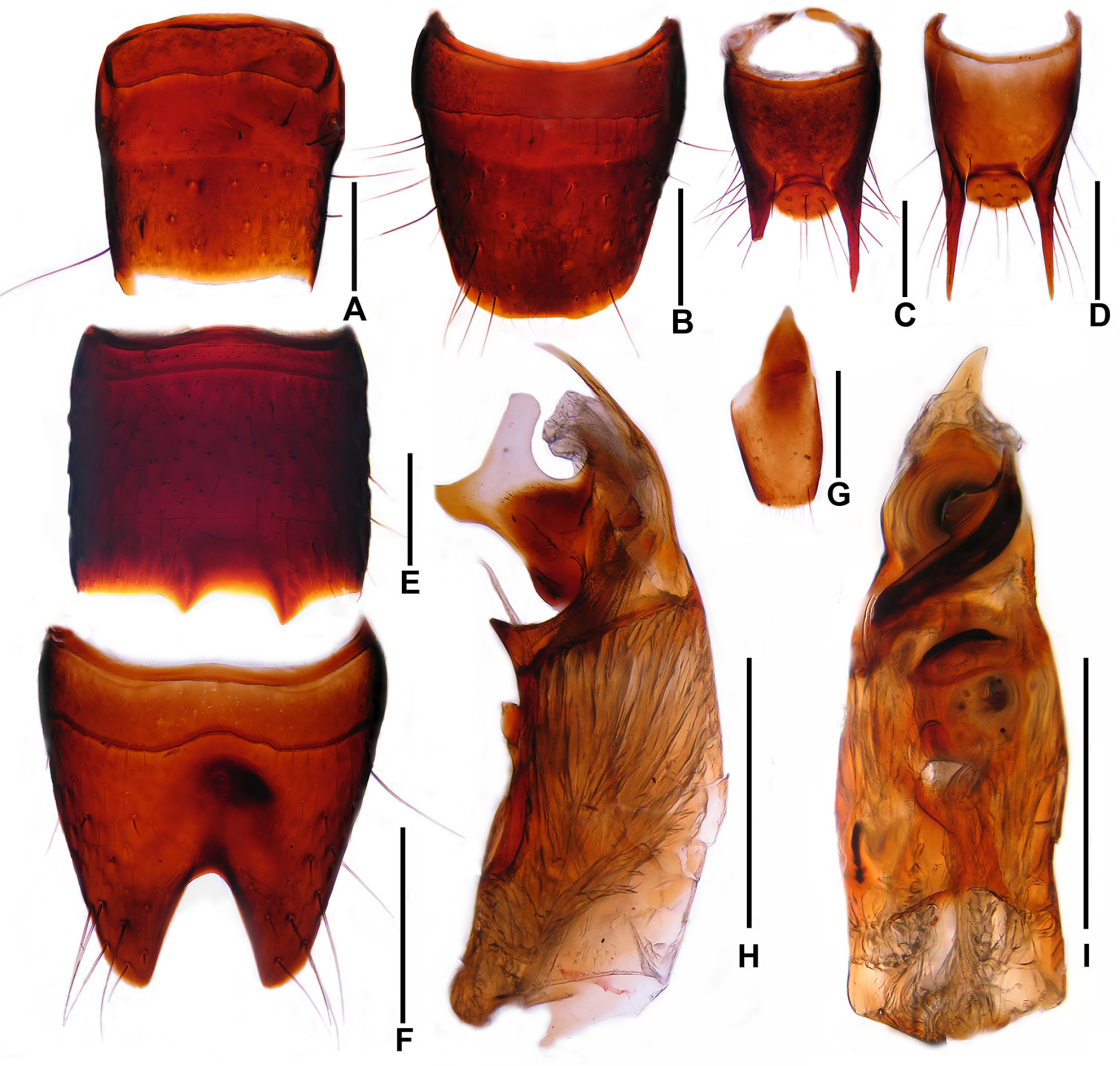
*Oedichirus acutus*. **A**. Female tergite VIII; **B**. Female sternite VIII; **C**. Female tergite IX; **D**. Male tergite IX; **E**. Male sternite VII; **F**. Male sternite VIII; **G**. Male sternite IX; **H**. Aedeagus in lateral view; **I**. Aedeagus in ventral view. Scale bars: 0.5 mm.

**Male**. Sternites III–VI unmodified. Sternite VII (Fig. 2E) weakly asymmetrical with shallow impunctate postero-median impression; posterior margin with angular projection on each side of midline. Sternite VIII (Fig. 2F) symmetrical, posterior excision deep and V-shaped. Tergite IX (Fig. 2D) with posterior processes 1.16–1.21 times as long as median portion of tergite IX. Sternite IX shaped as in Fig. 2G. Aedeagus (Figs 2H, 2I) asymmetrical; ventral process lamellate, and acute apically in lateral view, subapically with two conspicuous processes pointing ventrad. Parameres long and slender.

**Female**. Abdominal tergite VIII (Fig. 2A) oblong, with weakly convex posterior margin. Posterior margin of abdominal sternite VIII (Fig. 2B) broadly convex. Tergite IX shaped as in Fig. 2C.

#### Distribution.

The new species is known from several localities in the Maolan Nature Reserve, which is close to the border between Guizhou and Guangxi provinces. Although all confirmed records are from the Guizhou side, the species is also expected to occur in the mountains of Guangxi Province.

#### Etymology.

The epithet is derived from the Latin adjective “*acutus*” and refers to the acute apex of the aedeagus in ventral view.

#### Comparative notes.

Based on the uniformly black body and the shape of the male sternites VII–VIII, this species may be allied to *O. ovalisis* Duan, Yue & Li, 2024, from which it is distinguished by the lighter coloration of legs, the shape of the pronotum, the deeper posterior excision of the male sternite VIII and the lamellate ventral process of the aedeagus. For illustrations of *O. ovalisis* see Duan et al. (2024: fig. 2A–J) and Fig. 5.

### Oedichirus astoni

Taxon classificationAnimaliaColeopteraStaphylinidae

Rougemont, 2018

090E27D3-BE24-5B4F-8496-7700EF874238

#### Material examined.

China – **Guangdong** • 1 ♂; Shenzhen City, Mt. Qiniang, 22°32'28"N, 114°35'08"E, 45 m, 23.III.2019, Tang, Shuai, Xia, Zhao & Zhou leg. (SNUC).

#### Comment.

Previously, *O. astoni* was recorded in several regions of China, including Guangdong, Chongqing and Hong Kong (Gu and Chen 2026). For illustrations of *O. astoni* see Rougemont (2018b: fig. 2), Xu et al. (2020: fig. 1a–d) and Gu and Chen (2026: fig. 1A–E).

### Oedichirus biwenxuani

Taxon classificationAnimaliaColeopteraStaphylinidae

Tang & Peng sp. nov.

347365A1-CF84-526D-871A-CB5862F1E5B2

https://zoobank.org/3EAD2416-B0F3-4865-840F-306C3261185A

Figs 1B, 3

#### Type material.

***Holotype***. China – **Yunnan** • ♂; glued on a card with two labels as follows: “China: Yunnan Prov., Yingjiang Co., Nabang Town, 300 m, 23.V.2013, Wen-Xuan Bi leg.” “HOLOTYPE: *Oedichirus biwenxuani* Tang & Peng, 2026, new species.” [red handwritten label]; (SNUC).

#### Description.

Measurements (in mm) and ratios: BL: 7.89; FL: 3.44; HL: 0.82; HW: 1.19; AnL: 2.16; PL: 1.19; PW: 1.12; EL: 1.23; EW: 1.53; AL: 1.25; HW/HL: 1.45; HW/PW: 1.06; HL/PL: 0.69; PW/PL: 0.94; EL/PL: 1.03.

***Habitus*** as in Fig. 1B. Coloration: head and apex of abdomen black; antennae brown to light brown; pronotum and first four abdominal segments red; elytra black, with posterior third red; legs light brown.

***Head*** transverse, broadest across eyes. Lateral contours behind eyes converged sharply with basal angles distinct. Dorsal surface with coarse and sparse punctures. ***Eyes*** strongly convex, 2.56 times as long as distance from posterior margin of eye to posterior constriction.

***Pronotum*** approximately as broad as head, widest anteriorly and gently tapering posteriorly. Median dorsal portion convex, surface with coarse and sparse punctures, weakly larger and denser than those on head, longitudinally midline area of pronotum without punctures.

***Elytra*** nearly square, surface with moderately dense and coarse punctures, distinctly denser than those on head and pronotum. Humeral angles and wings well developed.

***Abdomen*** with dense punctures, arranged in rows; punctures on segments III–VI weakly denser than on segments VII–VIII. Interstices with fine and very shallow microsculpture; posterior margin of tergite VII with palisade fringe.

**Figure 3.**
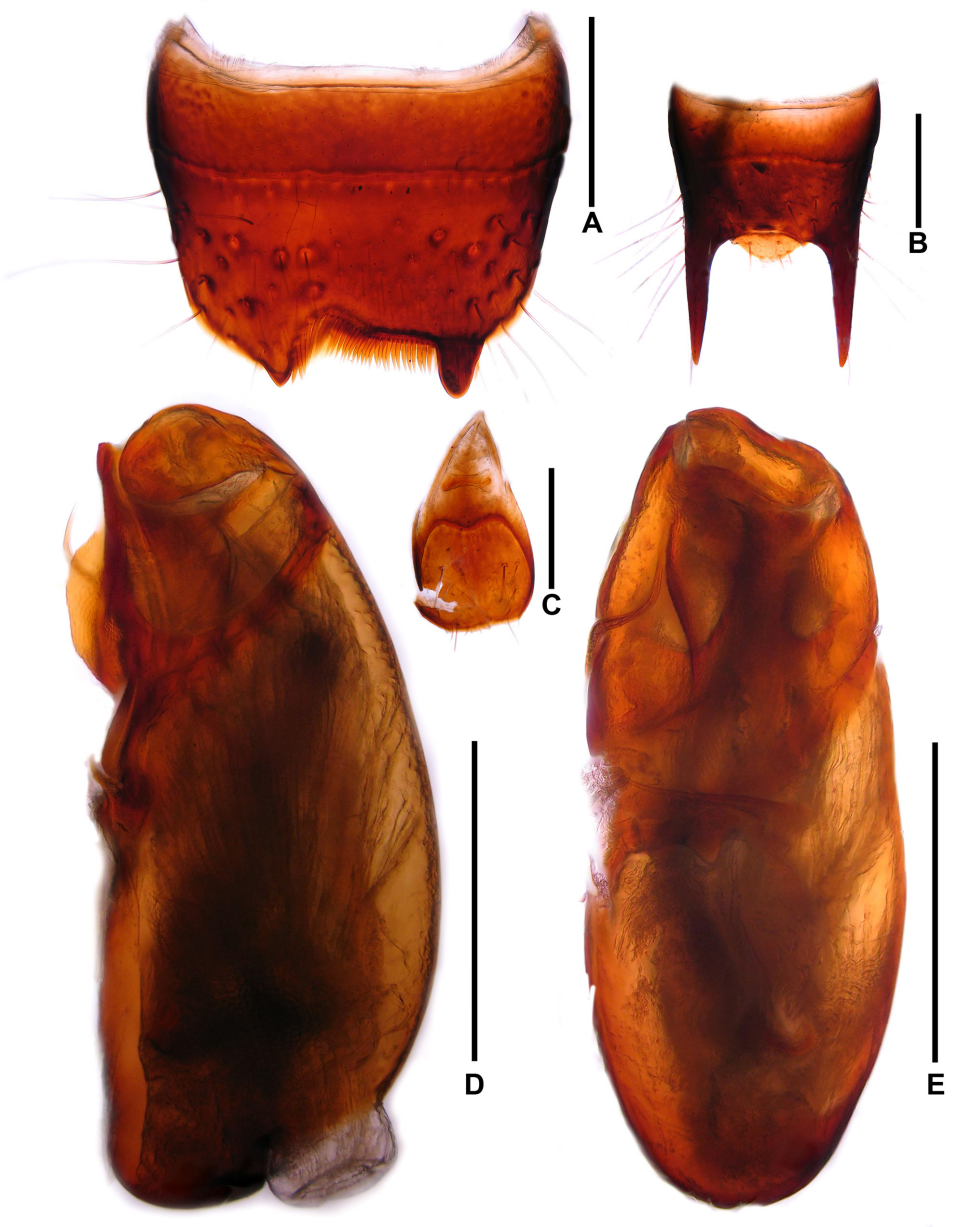
*Oedichirus biwenxuani*. **A**. Male sternite VIII; **B**. Male tergite IX; **C**. Male sternite IX; **D**. Aedeagus in lateral view; **E**. Aedeagus in ventral view. Scale bars: 0.5 mm.

**Male**. Sternites VI–VII unmodified. Sternite VIII (Fig. 3A) with conspicuously asymmetrical posterior excision and apical ctenidium composed of stout pectinate setae. Tergite IX (Fig. 3B) with posterior processes 1.03 times as long as median portion of tergite IX. Sternite IX shaped as in Fig. 3B. Aedeagus (Fig. 3D, E) nearly symmetrical; ventral process short and stout, subapically without conspicuous processes pointing ventrad. Parameres moderately long and slender.

**Female**. Unknown.

#### Distribution.

The species is currently known only from Nabang Town, located in western Yingjiang County, western Yunnan Province, China.

#### Etymology.

Named after Mr Wen-Xuan Bi (Shanghai, China), the collector of the holotype and a specialist on Cerambycidae.

#### Comparative notes.

Based on the coloration of the body, the morphology of the aedeagus and the male secondary sexual characters, *O. biwenxuani* is undoubtedly closely related to *O. sihanouki* Rougemont, 2018 and allied species distributed in the Oriental region, and attributed to the *O. alatus* group (Rougemont, 2018b). *Oedichirus biwenxuani* can be distinguished from *O. sihanouki* Rougemont, 2018 by the slenderer pronotum, the shape and chaetotaxy of the male sternite VIII, and the slenderer aedeagus in lateral view. For illustrations of *O. sihanouki* see Rougemont (2018b: fig. 8).

### Oedichirus chapmani

Taxon classificationAnimaliaColeopteraStaphylinidae

Cameron, 1940

7805C831-42BE-56EA-BC9A-04DCCFFA5582

#### Material examined.

China – **Hubei** • 1 ♂; Jianli City, Hewang Miao, 29°43'53"N, 113°01'30"E, 29 m, 24.IX.2020, Shen Zhao leg. (SNUC); China – **Guangdong** • 1 ♂; Shenzhen City, Mt. Qiniang, 22°32'28"N, 114°35'08"E, 45 m, 25.III.2019, Tang, Shuai, Xia, Zhao & Zhou leg. (SNUC); China – **Guangdong** • 1 ♀; Shenzhen City, Mt. Qiniang, 22°32'28"N, 114°35'08"E, 65 m, 05.VI.2019, Cai, Huang, Tang, Shuai & Zhao leg. (SNUC); China – **Yunnan** • 2 ♂♂; Nabanhe N. R., Naban Village, ca. 650 m, 07.I.2004, Li & Tang leg; (SNUC).

#### Comment.

Previously, this species was known from India, Bangladesh, Thailand, Vietnam, Laos, Japan, the Chinese province of Fujian, and Taiwan (Rougemont 2018b). The above specimens from Hubei, Guangdong and Yunnan represent new provincial records. For illustrations of *O. chapmani* see Rougemont (2018b: fig. 3).

### Oedichirus excisus

Taxon classificationAnimaliaColeopteraStaphylinidae

Tang & Peng sp. nov.

EB387740-5EBF-5482-8179-EA3EC2E6B1AC

https://zoobank.org/206D9122-9142-40AB-A81F-5DAD10AD558B

Figs 1C, 4

#### Type material.

***Holotype***. China – **Yunnan** • ♂; glued on a card with two labels as follows: “China: Yunnan Prov., Qujing City, Yong’an Village, 25.99°N, 104.07°E, 2143 m, 04.VII.2024, Huang, Jiang, Li & Tian leg.” “HOLOTYPE: *Oedichirus excisus* Tang & Peng, 2026, new species.” [red handwritten label]; (SNUC).

#### Description.

Measurements (in mm) and ratios: BL: 9.06; FL: 4.33; HL: 0.88; HW: 1.20; AnL: 3.30; PL: 1.34; PW: 1.04; EL: 1.60; EW: 1.54; AL: 1.08; HW/HL: 1.36; HW/PW: 1.15; HL/PL: 0.66; PW/PL: 0.78; EL/PL: 1.19.

***Habitus*** as in Fig. 1C. Coloration: body brown with dark apex; elytra brown, with almost all of posterior half completely blackish; antennae and legs yellowish brown.

***Head*** transverse, broadest across eyes. Lateral contours behind eyes converged sharply with basal angles distinct. Dorsal surface with dense and coarse punctures. Eyes convex, 2.04 times as long as distance from posterior margin of eye to posterior constriction.

***Pronotum*** weakly broader than head, widest anteriorly and gently tapering posteriorly. Median dorsal portion slightly convex, surface with dense and coarse punctures, distinctly denser and larger than those on head.

***Elytra*** oblong, surface with dense and coarse punctures, weakly denser than those on head and finer than pronotum. Humeral angles and wings well developed.

***Abdomen*** with dense punctures; punctures on segments III–VI distinctly coarser and denser than on segments VII–VIII. Interstices with fine and shallow microsculpture; posterior margin of tergite VII with palisade fringe.

**Figure 4.**
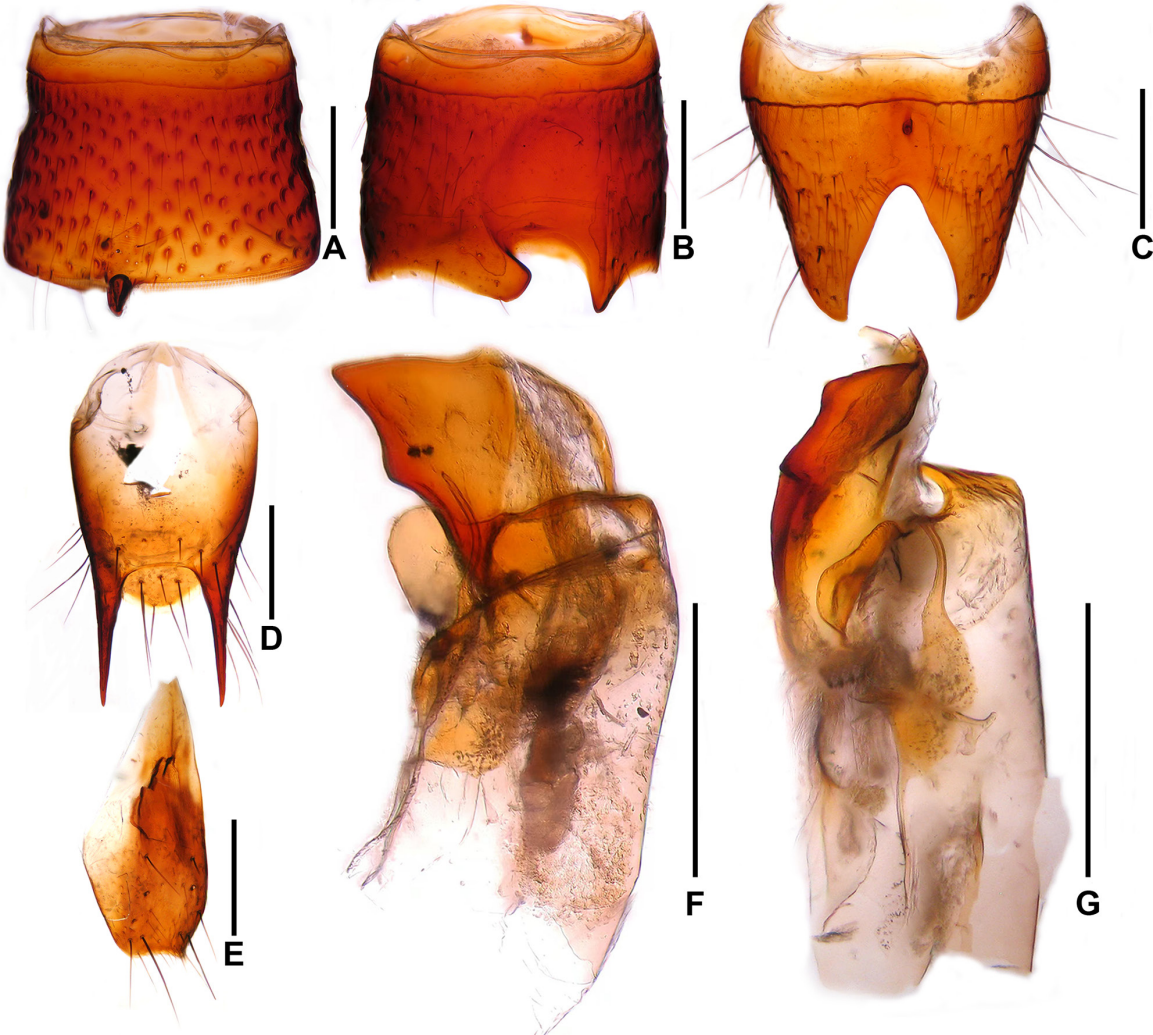
*Oedichirus excisus*. **A**. Male sternite VI; **B**. Male sternite VII; **C**. Male sternite VIII; **D**. Male tergite IX; **E**. Male sternite IX; **F**. Aedeagus in lateral view; **G**. Aedeagus in ventral view. Scale bars: 0.5 mm.

**Male**. Sternites III–V unmodified. Sternite VI (Fig. 4A) symmetrical, posterior margin with oxhorn-shaped projection. Sternite VII (Fig. 4B) asymmetrical, with deep and large impunctate postero-median impression; posterior margin with well-developed projection on each side of midline. Sternite VIII (Fig. 4C) symmetrical, subtriangular posterior excision relatively large and deep. Tergite IX (Fig. 4D) with posterior processes 1.06 times as long as median portion of tergite IX. Sternite IX shaped as in Fig. 4E. Aedeagus (Figs 4F, 4G) asymmetrical; ventral process large and flattened in lateral view, subapically with one conspicuous process. Parameres long and slender.

**Female**. Unknown.

#### Distribution.

The species is currently known only from Yong’an Village, located in northern Qujing City, northeastern Yunnan Province, China.

#### Etymology.

The specific epithet is the superlative of the Latin adjective *excisus* (excised) and alludes to the conspicuously deep posterior excision of the male sternite VIII.

#### Comparative notes.

As can be inferred from the similar male sexual characters, *O. excisus* is a close relative of *O. tibetanus* Duan, Yue & Li, 2024. It is distinguished from the latter by the coloration of the body, the shorter oxhorn-shaped projection on the posterior margin of the male sternite VI, as well as by the different shape of the ventral process of the aedeagus. For illustrations of *O. tibetanus* see Duan et al. (2024: fig. 3A–K).

### Oedichirus laoticus

Taxon classificationAnimaliaColeopteraStaphylinidae

Rougemont, 2018

0A8EBD1D-910F-5C4D-90AE-96FB53C3AF14

#### Material examined.

China – **Guangxi** • 1 ♂; Shangsi Co., Shiwandashan N. R., 21.85°N, 107.89°E, 523 m, 24.VIII.2024, Huang, Jiang, Li, Lin & Liu leg. (SNUC).

#### Comment.

The species has been described from northeastern Laos. The newly recorded locality in Guangxi is situated approximately 420 km from the type locality of *O. laoticus*, and represents the first record of this species from China. For illustrations of *O. laoticus* see Rougemont (2018b: fig. 46).

### Oedichirus longipennis

Taxon classificationAnimaliaColeopteraStaphylinidae

Kraatz, 1859

D738BF5B-8429-5CA5-81C8-B7D59095F2F1

#### Material examined.

China – **Shanghai** • 1 ♂, 1 ♀; Chongming, 08, 10.VIII.2007, Jiao-Yao Hu leg. (SNUC); China – **Shanghai** • 1 ♂; Chongming, 27.IX.2009, Jiao-Yao Hu leg. (SNUC); China – **Zhejiang** • 8 ♂♂, 4 ♀♀; Zhuji City, Caota Town, 100 m, 20.XI.2011, Tie-Xiong Zhao leg. (SNUC); China – **Zhejiang** • 1 ♂; Jinhua City, Panan Co., Majian Town, 100 m, 15.II.2014, Tie-Xiong Zhao leg. (SNUC); China – **Zhejiang** • 1 ♀; Linan City, West Tianmushan, 30°24'22"N, 119°27'41"E, 950–1000 m, 07.IX.2024, Ding, Yin & Zhang leg. (SNUC); China – **Guangdong** • 3 ♂♂, 1 ♀; Shenzhen City, Mt. Qiniang, 22°32'28"N, 114°35'08"E, 63 m, 15.XI.2018, Cheng, Peng, Shuai & Zhao leg. (SNUC); China – **Guangdong** • 6 ♂♂; Shenzhen City, Mt. Qiniang, 22°32'28 "N, 114°35'08"E, 45 m, 23.III.2019, Tang, Shuai, Xia, Zhao & Zhou leg. (SNUC); China – **Guangdong** • 1 ♂; Shenzhen City, Mt. Wutong, 22°34'53"N, 114°36'49"E, 590 m, 11.VII.2019, Chang, Xia, Zhao & Zhou leg. (SNUC); China – **Guangdong** • 1 ♀; Shenzhen City, Mt. Wutong, 22°34'53"N, 114°36'48"E, 590 m, 11.VII.2019, Chang, Xia, Zhang, Zhao & Zhou leg. (SNUC); China – **Guangdong** • 1 ♂; Guangzhou City, Haizhu Wetland, 23°04'32"N, 113°18'24"E, 11.X.2020, Jiang Zhu leg. (SNUC); China – **Guangxi** • 1 ♂, 1 ♀; Huanjiang Co., Mulun N. R., 25°07'08"N, 107°58'33"E, 550 m, 27.IV.2021, Cai, Peng, Song & Tang leg. (SNUC); China – **Hainan** • 3 ♂♂, 2 ♀♀; Yinggeling N. R., 26.XII.2005, Kai Nan leg. (SNUC); China – **Hainan** • 1 ♂; Yinggeling N. R., 800 m, 20.VII.2009, Zhu-Qing He leg. (SNUC); China – **Hong Kong** • 1 ♂; Mt. Taimo, 22°24'59"N, 114°07'37"E, 774 m, 14.V.2018, Zhong Peng leg; (SNUC).

#### Comment.

This species was previously recorded from Pakistan, India, Nepal, Thailand, Laos, Vietnam, Sri Lanka, Malaysia, Singapore, Indonesia and Japan, as well as from several regions of China, including Shanghai, Guangxi, Yunnan, Hong Kong and Taiwan (Rougemont 2018b). It is among the most widely distributed species in the genus. The above specimens from Zhejiang, Guangdong and Hainan represent new provincial records. For illustrations of *O. longipennis* see Rougemont (2018b: fig. 14).

### Oedichirus ovalisis

Taxon classificationAnimaliaColeopteraStaphylinidae

Duan, Yue & Li, 2024

9BAB0970-F2ED-5FA3-AF3A-4E93B8B61425

Fig. 5

#### Material examined.

China – **Guangxi** • 1 ♂; Huanjiang Co., Mulun N. R., 25°03'12"N, 107°57'59"E, 450–650 m, 26.VII.2015, Chen, He & Hu leg. (SNUC); China – **Guangxi** • 1 ♂; Debao Co., ca. Yibei Village, 23.34°N, 106.69°E, 714 m, 30.VII.2024, Huang, Jiang, Li & Tian leg. (SNUC); China – **Guangxi** • 1 ♀; Napo Co., Pingmeng Town, Meilin Shan, 22°58'51"N, 105°50'36"E, 1136 m, 17.VII.2024, Qian-Le Lu leg. (SNUC); China – **Hainan** • 2 ♂♂, 1 ♀; Ledong Co., Jianfengling, Mingfenggu, 18°44'N, 108°50'E, 950–1000 m, 11–23.V.2011, Wen-Xuan Bi leg. (SNUC).

#### Comment.

**Figure 5.**
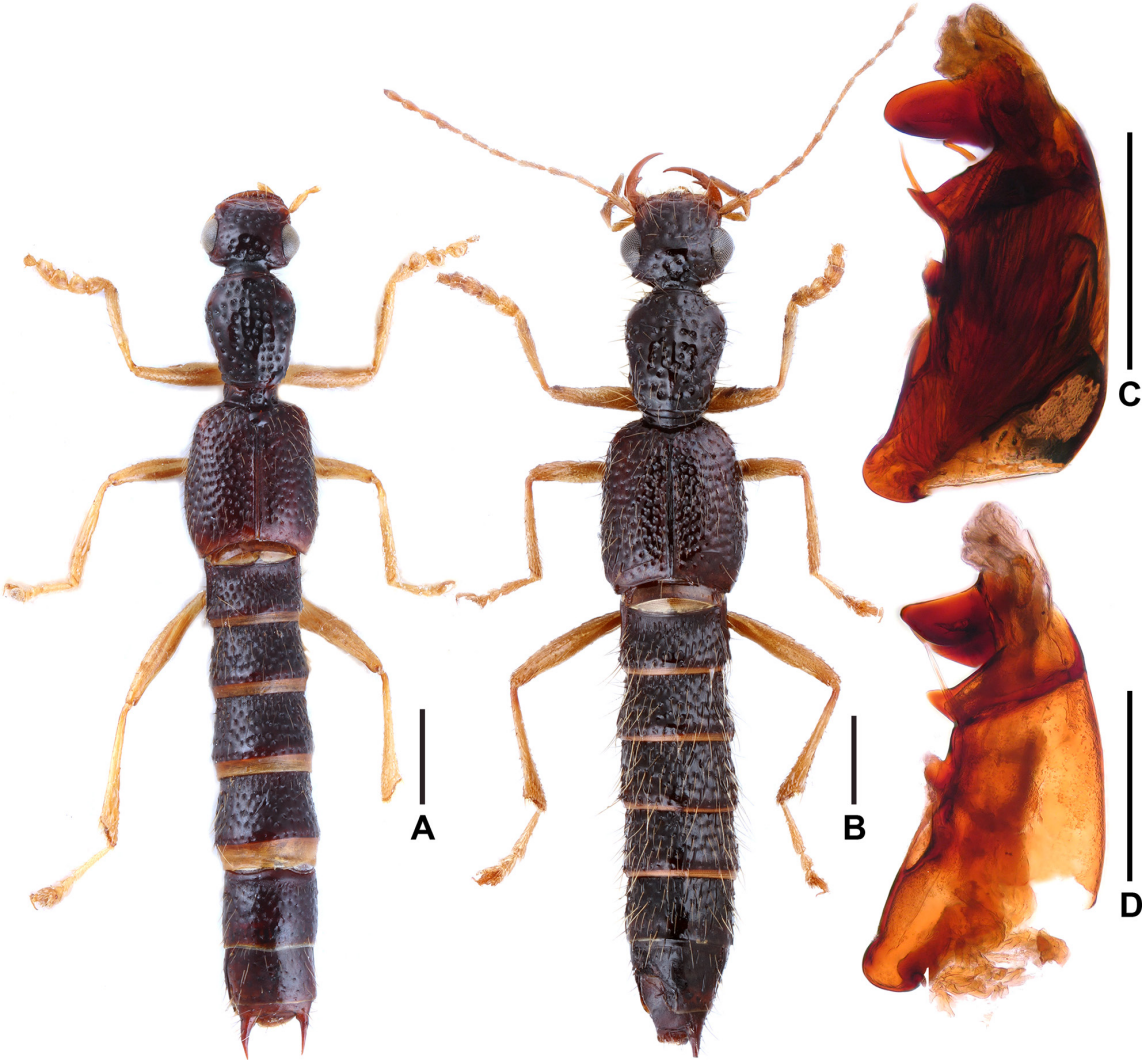
*Oedichirus ovalisis*. **A**. Habitus (from Hainan); **B**. Habitus (from Guangxi); **C**. Aedeagus in lateral view (from Hainan); **D**. Aedeagus in lateral view (from Guangxi). Scale bars: 1 mm (**A, B**); 0.5 mm (**C, D**).

*Oedichirus ovalisis* was previously known from the Chinese provinces of Fujian and Hainan (Duan et al. 2024). Examination of the material at hand reveals considerable intraspecific variation in morphological traits, including body size, coloration, punctation, and the shapes of the head, pronotum and elytra (Fig. 5A, B). In contrast, the shape of male sternites VII–VIII remains constant, and the aedeagus exhibits minimal variation (Fig. 5C, D). The above records from Guangxi represent new province records.

### Oedichirus tibetanus

Taxon classificationAnimaliaColeopteraStaphylinidae

Duan, Yue & Li, 2024

8924C591-04CD-5FAC-95BD-C6022B8E3A8B

#### Material examined.

China – **Xizang** • 2 ♂♂, 2 ♀♀; Medog Co., Beibeng, 800–1000 m, 01.VIII.2014, Wen-Xuan Bi leg. (SNUC).

#### Comment.

This species was only recently described from Medog County, southeast Xizang (Duan et al. 2024).

##### Key to the macropterous species of *Oedichirus* from China

In the key published by Duan et al. (2024), seven macropterous species of *Oedichirus* were included, among which two species from Taiwan, *O. formosanus* Rougemont, 2018 and *O. guomindangi* Rougemont, 2018 (both described based on single female specimens), were not covered. Additionally, *O. lewisius* Sharp, 1874 was misidentified and should be excluded from the key. This paper presents taxonomic and faunistic data for nine macropterous species, including three new species. Accordingly, an updated key to the macropterous species of China is provided.

**Table:** 

1	Coloration of head and pronotum different (head black and pronotum red)	**2**
–	Coloration of head and pronotum uniform (reddish brown to black)	**6**
2	Elytra unicolorous, black; antenna shorter (AnL 1.65 mm)	***O. longipennis* Kraatz, 1859**
–	Elytra bicolorous, black apically, and with half or posterior third red; antenna longer (AnL ≥ 1.8 mm)	**3**
3	Abdominal segments III–V red; posterior halves of meso- and metafemora of legs light brown, distal halves gradually infuscate; length of forebody (FL): 3.1 mm	**4**
–	Abdominal segments III–VI red; legs unicolorous, light brown or testaceous; length of forebody (FL) more than 3.4 mm	**5**
4	Head more transverse (HW/HL 1.31); elytra width (EW): 1.3 mm	***O. astoni* Rougemont, 2018**
–	Head less transverse (HW/HL 1.21); elytra width (EW): 1.42 mm	***O. guomindangi* Rougemont, 2018**
5	Larger species (BL 7.89 mm, FL 3.44 mm); antenna much longer (AnL 2.16 mm); aedeagus stouter, without conspicuous processes in lateral view	***O. biwenxuani* Tang & Peng, sp. nov**.
–	Smaller species (BL 7 mm, FL 3.4 mm); antenna much shorter (AnL 1.8 mm); aedeagus slenderer, with two conspicuous processes in lateral view	***O. chapmani* Cameron, 1940**
6	Coloration of body lighter, reddish brown or brown, but with dark or black apex	**7**
–	Coloration of body much darker, uniformly black	**8**
7	Pronotum stouter (PW/PL: 0.78); posterior margin of male sternite VI with oxhorn-shaped projection; male sternite VII asymmetrical, with deep and large postero-median impression; male sternite VIII with symmetrical subtriangular posterior excision	***O. excisus* Tang & Peng, sp. nov**.
–	Pronotum slenderer (PW/PL: 0.73); posterior margin of male sternite VI without projection; male sternite VII symmetrical, without conspicuous impression; male sternite VIII with asymmetrical posterior excision	***O. kuroshio* Hayashi, 1989**
8	Larger species (BL ≥ 11.5 mm); elytra width (EW) more than 1.75 mm	**9**
–	Smaller species (BL ≤ 10.6 mm); elytra width (EW) no more than 1.60 mm	**10**
9	Head width (HW): 1.25 mm; punctures on disc of pronotum distinctly arranged in series	***O. formosanus* Rougemont, 2018**
–	Head width (HW): 1.82 mm; punctures on disc of pronotum not forming discernible series	***O. laoticus* Rougemont, 2018**
10	Pronotum much longer (PL: 1.44 mm); posterior margin of male sternite VI with sharply pointed keel; posterior excision of male sternite VIII deeper; aedeagus with one process subapically	***O. tibetanus* Duan, Yue & Li, 2024**
–	Pronotum shorter (PL ≤ 1.26 mm); posterior margin of male sternite VI without pointed keel; posterior excision of male sternite VIII shallower; aedeagus with two processes subapically	**11**
11	Legs yellowish brown; pronotum longer (PL: 1.15–1.26 mm); male sternite VIII with deeper posterior excision; ventral process of aedeagus lamellate	***O. acutus* Tang & Peng, sp. nov**.
–	Legs brown; pronotum shorter (PL: 1.0 mm); male sternite VIII with shallower posterior excision; ventral process of aedeagus stout	***O. ovalisis* Duan, Yue & Li, 2024**

## Supplementary Material

XML Treatment for Oedichirus acutus

XML Treatment for Oedichirus astoni

XML Treatment for Oedichirus biwenxuani

XML Treatment for Oedichirus chapmani

XML Treatment for Oedichirus excisus

XML Treatment for Oedichirus laoticus

XML Treatment for Oedichirus longipennis

XML Treatment for Oedichirus ovalisis

XML Treatment for Oedichirus tibetanus

## Additional information

### Artificial Intelligence (AI) use

The authors accept full responsibility for the content of the manuscript, including the disclosure of any use of AI.

No AI tools were used in the preparation of this manuscript.

### Author ORCIDs

S.-C. Tang https://orcid.org/0009-0005-9791-8671

Z. Peng https://orcid.org/0000-0001-5959-1536

### Data availability

All of the data that support the findings of this study are available in the main text.

## References

[B1] Assing V (2014) On the *Oedichirus* fauna of China (Coleoptera: Staphylinidae: Paederinae). Linzer biologische Beiträge 46(2): 1229–1240. 10.5281/zenodo.5308780

[B2] Assing V (2019) Three new species and additional records of *Oedichirus* (Coleoptera: Staphylinidae: Paederinae). Linzer biologische Beiträge 51(1): 33–42. 10.5281/zenodo.3763597

[B3] Duan JD, Yue CM, Ma HY, Lin HQ, Li XY (2024) Three new species of the genus *Oedichirus* Erichson, 1839 (Coleoptera: Staphylinidae: Paederinae: Pinophilini) from China. Zootaxa 5528(1): 383–392. 10.11646/zootaxa.5528.1.28 39646880

[B4] Gu TX, Chen JH (2026) New record of *Oedichirus astoni* (Coleoptera: Staphylinidae: Paederinae) in Chongqing. The Indochina Entomologist 2(12): 131–134. 10.70590/ice.2026.02.12

[B5] Li WR, Xie NN, Li LZ (2015) Redescription of *Oedichirus flammeus* Koch, and description of two new *Oedichirus* species from China (Coleoptera, Staphylinidae, Paederinae, Pinophilini). Zootaxa 3911(1): 81–90. 10.11646/zootaxa.3911.1.4 25661597

[B6] Rougemont GM de (2018a) New Papuan *Oedichirus* (Staphylinidae, Paederinae, Pinophilini). Linzer Biologische Beiträge 50(1): 435–446. 10.5281/zenodo.5779806

[B7] Rougemont GM de (2018b) New oriental *Oedichirus* (Staphylinidae, Paederinae, Pinophilini). Linzer Biologische Beiträge 50(1): 461–536. 10.5281/zenodo.4004245

[B8] Xu W, Liang H, Tang L, Zeng Q, Qiao YJ (2020) New records of *Oedichirus astoni* and *Oedichirus lewisii* (Coleoptera: Staphylinidae: Paederinae). Journal of East China Normal University (Natural Science) 2020(6): 140–143. [in Chinese, with English title and abstract] 10.3969/j.issn.1000-5641.201932007

